# Ventriculosubgaleal shunt placement for hydrocephalus in osteogenesis imperfecta with novel compound heterozygous *CRTAP* variants

**DOI:** 10.1038/s41439-024-00274-z

**Published:** 2024-03-28

**Authors:** Shintaro Nakamura, Kyosuke Ibi, Hiroyuki Tanaka, Hirokazu Takami, Keita Okada, Nao Takasugi, Motohiro Kato, Naoto Takahashi, Takanobu Inoue

**Affiliations:** 1 Department of Pediatrics, The University of Tokyo, Tokyo, Japan; 2 Department of Neurosurgery, The University of Tokyo, Tokyo, Japan; 3 Department of Orthopaedic Surgery, The University of Tokyo, Tokyo, Japan

**Keywords:** Osteogenesis imperfecta, Medical genetics

## Abstract

Osteogenesis imperfecta is characterized by frequent fractures, bone deformities, and other systemic symptoms. Severe osteogenesis imperfecta may progress to hydrocephalus; however, treatment strategies for this complication remain unclear. Here, we describe severe osteogenesis imperfecta in an infant with symptomatic hydrocephalus treated with ventriculosubgaleal shunt placement. Targeted next-generation sequencing revealed novel compound heterozygous *CRTAP* variants, i.e., NM_006371.5, c.241 G > T, p.(Glu81*) and NM_006371.5, c.923-2_932del. We suggest that ventriculosubgaleal shunt placement is an effective and safe treatment for hydrocephalus in patients with severe osteogenesis imperfecta.

Osteogenesis imperfecta (OI) is a phenotypically and genetically heterogeneous connective tissue disorder characterized by symptoms such as a high frequency of fractures, bone deformities, growth deficiency, blue sclerae, hearing loss, and decreased pulmonary function. The Sillence classification system divides OI into four types (I [mild], II [lethal], III [severe], and IV [moderate]) based on its clinical and radiographic features^1^. Severe OI may also involve hydrocephalus;^2^ however, treatment strategies for this condition remain unclear.

Approximately 85–90% of cases of OI are caused by pathogenic variants in *COL1A1* and *COL1A2*, which encode the α1 (I) and α2 (I) chains of type I collagen, respectively. In addition, genes involved in the processing, posttranslational modification, folding, and cross-linking of type I collagen have been identified to cause OI. Cartilage-associated protein (CRTAP) is involved in the posttranslational modification and folding of type I collagen^1^. Biallelic loss-of-function *CRTAP* variants lead to Sillence type II, III, or IV OI^3^. Here, we report a case of severe OI in an infant with symptomatic hydrocephalus harboring novel compound heterozygous *CRTAP* variants and requiring ventriculosubgaleal (VSG) shunt placement.

A 33-year-old primigravida Japanese woman (I-2 in Fig. 1a) was referred to our hospital after short and incurved fetal limbs were detected during routine prenatal ultrasonography at 22 weeks of gestation. She and her husband (I-1) were nonconsanguineous with no remarkable personal or family history, including bone disorders. A prenatal three-dimensional computed tomography (CT) scan performed at 31 weeks of gestation revealed decreased fetal ossification of the skull, deformities and fractures of the ribs and long bones, and a small thorax (Fig. 1b). The female infant (II-1) was born via breech vaginal delivery at 38 weeks of gestation with 1- and 5-min Apgar scores of 4 and 6, respectively. Her birth length was 50.0 cm (−0.7 standard deviation [SD]), and her birth weight was 2,677 g (−0.5 SD). The patient did not present with blue sclerae. The patient was immediately intubated due to pulmonary hypoplasia. In addition, she required therapy for low blood pressure and disseminated intravascular coagulation due to bleeding from long bone fractures. Radiography at birth demonstrated the presence of Wormian bones in the skull and selective shortening of the humeri and femora (Fig. 1c). The patient was diagnosed with severe OI. Intravenous pamidronate treatment was initiated on Day 40 and repeated every 2 months.

**Fig. 1:**
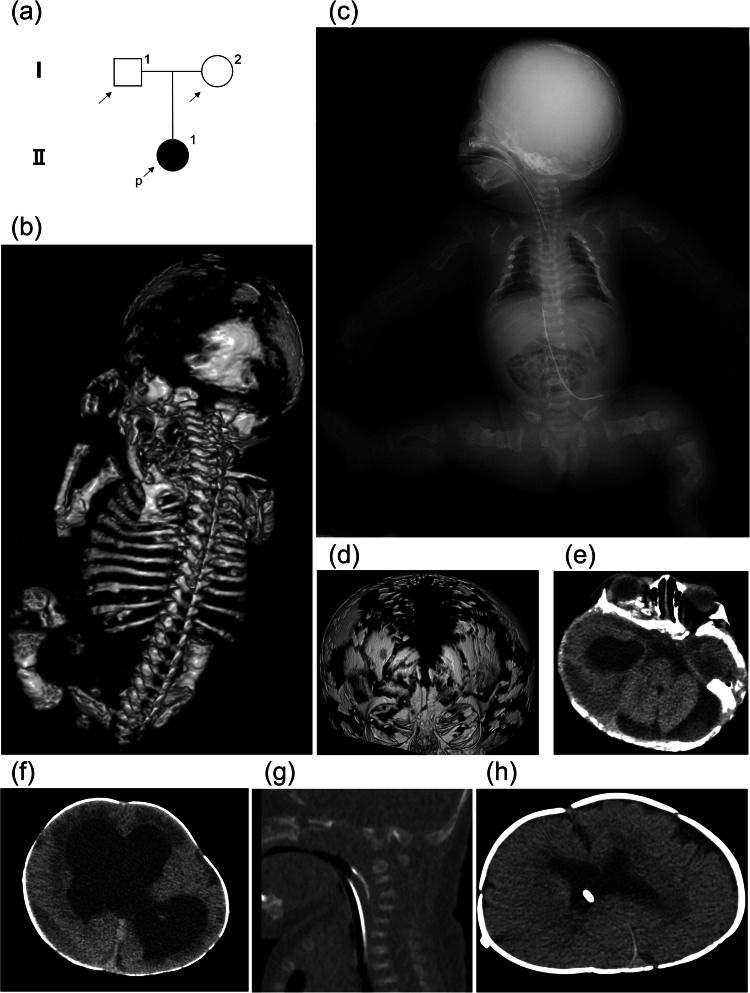
Clinical findings of the patient. **a** Pedigree of the family. **b** Three-dimensional computed tomography (CT) image taken at 31 weeks of gestation. **c** Systemic radiography at birth showing the Wormian bones in the skull, deformities and fractures of the ribs and long bones, selective shortening of the humeri and femora, and a bell-like thorax. **d** Three-dimensional CT image on Day 25 showing an extremely thin skull. **e** Axial CT image on Day 25 showing a chronic subdural hematoma in the posterior cranial fossa. **f** Axial CT image on Day 25 showing ventriculomegaly and a chronic subdural hematoma in the right temporal region. **g** Sagittal image on Day 25 showing instability of the craniovertebral junction. **h** Axial CT image on Day 287 showing no ventriculomegaly after ventriculoperitoneal shunt surgery.

Although neither ventricular dilatation nor intraventricular hemorrhage was detected at birth, we observed ventricular dilatation via cranial ultrasonography on Day 3 postpartum. Progressive macrocephaly and sunset eyes were observed. On Day 25, CT images revealed fragmented features of the skull, chronic subdural hematoma in the posterior cranial fossa and right temporal region that presumably occurred during delivery, severe ventriculomegaly, and instability of the craniovertebral junction (Fig. 1d-g). Ventriculoperitoneal (VP) shunt placement was difficult because appropriate head and neck positioning and tunneling of the device over the vulnerable skull, cervical spine, and thorax posed significant challenges at this stage. Consequently, on Day 35, she underwent VSG shunt placement as a temporary measure to treat her hydrocephalus. The patient was extubated on Day 43. After allowing time for bone maturation, on Day 96, the patient underwent VP shunt placement as the definitive surgery. No major bone fractures were observed during or after the surgery. Postoperatively, the ventriculomegaly resolved (Fig. 1h). Despite being unable to track objects with her eyes preoperatively, she developed eye tracking at approximately 5 months of age, displayed a social smile at approximately 7 months of age, and spoke one word at approximately 11 months of age. The patient was discharged at 11 months of age. At the latest follow-up at 18 months of age, she used supplemental low-flow oxygen while sleeping because of pulmonary hypoplasia and airway obstruction. She exhibited moderate bilateral hearing loss and was treated with hearing aids. Although she was bedridden and her motor development was delayed, as evidenced by poor head control and rolling ability, she gained the ability to express several one-word utterances and ingest baby food orally. Moreover, she was fed milk using a nasoduodenal tube to prevent gastroesophageal reflux. She was 60.0 cm (−7.1 SD) in length and 5,956 g (−5.6 SD) in weight. Based on her clinical course, she was diagnosed with type III OI according to the Sillence classification^1^.

After we obtained written informed consent from the patient’s parents in accordance with the Institutional Review Board Committee of the University of Tokyo, we performed targeted next-generation sequencing of the proband as previously reported^4^. We screened for nine OI-causative genes: *BMP1*, *COL1A1*, *COL1A2*, *CRTAP*, *FKBP10*, *IFITM5*, *P3H1*, *PPIB*, and *SERPINF1*. Allele frequencies were evaluated based on the Genome Aggregation Database, Human Genetic Variation Database, Japanese Multi Omics Reference Panel, and GEnome Medical alliance Japan Whole Genome Aggregation Panel. Common variants (with an allele frequency >0.005) were excluded. Validation and segregation analyses of the identified variants were performed by Sanger sequencing. We conducted a pathogenicity assessment of rare variants based on the American College of Medical Genetics and Genomics (ACMG) guidelines^5^ and extracted “pathogenic” or “likely pathogenic” variants. To predict the pathogenicity of rare variants, we conducted in silico analyses using Combined Annotation Dependent Depletion and MutationTaster.

We identified two novel *CRTAP* variants in the patient. One nonsense variant (NM_006371.5, c.241 G > T, p.(Glu81*)) was inherited from her father, while a deletion variant (NM_006371.5, c.923-2_932del) encompassing the splicing acceptor site of intron 4 and exon 5 was inherited from her mother (Fig. 2a). According to the ACMG guidelines,^5^ both variants were classified as “pathogenic” (Fig. 2b).

**Fig. 2:**
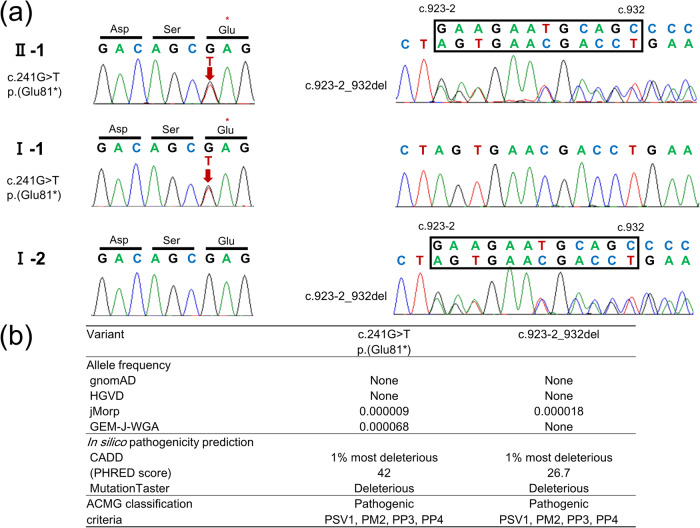
Pathogenic *CRTAP* variants detected in this study. **a** Chromatograms of variants in the infant and her parents. **b** Allele frequencies and in silico pathogenicity predictions of the variants. The accession number is *CRTAP* NM_006371.5. gnomAD Genome Aggregation Database, HGVD Human Genetic Variation Database, jMorp Japanese Multi Omics Reference Panel, GEM-J-WGA GEnome Medical alliance Japan Whole Genome Aggregation Panel, CADD Combined Annotation Dependent Depletion, ACMG American College of Medical Genetics and Genomics.

Both variants detected in the patient occurred at extremely low frequencies in the general Japanese population (Fig. 2b). The first variant was a nonsense variant located in exon 1, which was predicted to undergo nonsense-mediated mRNA decay. Although the other variant was a deletion variant not previously reported in patients with OI, a similar variant located in the splicing acceptor site of intron 4 (NM_006371.5, c.923-2 A > G) was reported in Sillence type III OI^6^. Our patient demonstrated a severe phenotype with selective shortening of the humeri and femora, similar to previously reported patients with OI harboring *CRTAP* variants^1^.

Although hydrocephalus is a known complication of severe OI,^2^ patients with OI harboring pathogenic *CRTAP* variants and hydrocephalus have not been reported previously. The possible mechanisms of hydrocephalus in patients with OI include basilar invagination, intracranial hemorrhage, primary disruption of cerebrospinal fluid (CSF) flow dynamics, and craniometric disproportion^2^ The exact developmental mechanism of hydrocephalus in the present case was unclear; no evidence of basilar invagination was present, but signs of intracranial hemorrhage and craniometric disproportion, such as instability of the craniovertebral junction, were observed. The intracranial hemorrhage was believed to be the major contributing factor to hydrocephalus in this case. The influx of hemorrhage into the subarachnoid space might have compromised the absorption capacity of the CSF. Moreover, the CSF outflow might have been partially obstructed due to the subdural hematoma and instability of the craniovertebral junction.

Here, wereport the first case of a patient with OI treated with VSG shunt placement for hydrocephalus. Although treatment strategies for hydrocephalus in patients with OI and extremely vulnerable bone structures remain unclear, there are some reports on surgical approaches used in early infancy. One report described a patient with Sillence type II OI who underwent endoscopic third ventriculostomy for noncommunicating hydrocephalus at 15 months of age^7^ Another report described a 14-month-old patient with Sillence type II OI and hydrocephalus who was treated with a VP shunt^8^. To our knowledge, our patient received the earliest surgical intervention for hydrocephalus among reported infants with OI. In contrast to VP shunt placement, VSG shunt placement is a less invasive but temporary option that is known to be useful for premature infants with posthemorrhagic hydrocephalus^9^. The best management scheme for this patient was VSG shunt placement, waiting for bone maturation with pamidronate therapy, and VP shunt placement.

Two additional points should be mentioned. First, we chose to deliver the patient vaginally. According to the largest analysis to date, cesarean delivery of patients with OI in breech position is not associated with a decreased rate of fractures presumably sustained during delivery^10^. Another study reported that cesarean delivery did not prolong the survival of patients with lethal OI^11^. Considering the maternal complications of cesarean delivery, we attempted vaginal delivery after obtaining informed consent from the parents. Second, we administered pamidronate to the present patient, who had multiple fractures, on Day 40. Although the ability of bisphosphonates to prevent fractures is unclear, there is sufficient evidence of their ability to increase bone mineral density in patients with OI^12^. We employed all possible methods to strengthen her bones prior to VP shunt placement. Moreover, the common concern about delayed fracture healing associated with bisphosphonate use did not outweigh its necessity in the present patient, since this concern has not been sufficiently evaluated according to the latest systematic review^12^.

In conclusion, we reported a case of severe OI complicated by hydrocephalus that was treated with early VSG shunt placement. We suggest that VSG shunt placement is an effective and safe approach for the treatment of symptomatic hydrocephalus in patients with severe OI during early infancy.

## HGV datbase

The relevant data from this Data Report are hosted at the Human Genome Variation Database at 10.6084/m9.figshare.hgv.3385. 10.6084/m9.figshare.hgv.3388.
